# Multiple Allergen Simultaneous Test-Immunoblot Assay for Immunoglobulin E Detection in Patients with Isolated Allergic Conjunctivitis

**DOI:** 10.3390/jcm10050960

**Published:** 2021-03-01

**Authors:** Jung Yeob Han, Hun Lee, Jae Lim Chung, Young Jun Kim, Jae Yong Kim, Hungwon Tchah

**Affiliations:** 1 Department of Ophthalmology, Asan Medical Center, University of Ulsan College of Medicine, Seoul 05505, Korea; dryeob@gmail.com (J.Y.H.); yhun777@gmail.com (H.L.); 2 Eyejun Ophthalmic Clinic, Seoul 06232, Korea; jaelim.chung@gmail.com (J.L.C.); einsjun@hanmail.net (Y.J.K.)

**Keywords:** allergic conjunctivitis, multiple allergen simultaneous test-immunoblot assay, serum immunoglobulin E

## Abstract

We aimed to investigate the immunoglobulin E (IgE) detection rate and allergen patterns in patients with isolated allergic conjunctivitis using the multiple allergen simultaneous test (MAST)-immunoblot assay. A total of 120 patients with allergic conjunctivitis and no associated rhinitis, asthma, or dermatitis underwent the MAST-immunoblot assay to measure serum total IgE (TIgE) and serum specific IgE (SIgE) against 57 allergens. Patients were classified into subgroups based on the season when the eye symptoms were exacerbated, and TIgE and SIgE positivity. Differences between sex and age groups were also analyzed. Of the 120 patients, 57.5% (69 patients) and 69.2% (83 patients) were positive for TIgE (≥100 IU/mL) and SIgE (≥0.7 IU/mL), respectively. The allergens that most frequently triggered sensitization in the study population were *Dermatophagoides farinae*, *Dermatophagoides pteronyssinus*, *Tyrophagus putrescentiae*, *Alternaria*, and house dust. House dust mites, such as *D. farinae* and *D. pteronyssinus*, showed the highest detection rates regardless of the season. Men had a higher positive rate for TIgE than women, whereas a higher rate of sensitization, detected as SIgE positivity, was seen in younger patients. In conclusion, MAST-immunoblot assay can detect sensitizing allergens in patients with isolated allergic conjunctivitis.

## 1. Introduction

Allergy is defined as type Ⅰ hypersensitivity reaction to certain substances known as allergens (e.g., environmental factors, food, and drugs) [1]. Approximately 15–20% of the world’s population develops a form of allergic disease, of which 40–60% of individuals are estimated to present ocular allergic symptoms [2]. Although ocular allergies are generally recognized as a disease accompanying allergic rhinitis, and many studies have been conducted on allergic rhinoconjunctivitis, isolated ocular allergies remain underestimated and are increasingly being recognized as an important independent disease [3]. In addition, ocular allergies are considered a major public health concern, leading to lower productivity and education efficiency [4]. 

Allergic diseases are usually diagnosed based on the clinical history and characteristic signs and symptoms; however, measuring immunoglobulin E (IgE) levels using in vivo or in vitro methods is performed to detect sensitization [5]. IgE plays an important role in the pathogenesis of allergy, and various methods of detecting IgE have been the subject of research [6]. Allergic reaction occurs in two pathophysiological steps. An initial sensitization phase is characterized by exposure to an allergen that is recognized by serum specific IgE (SIgE) antibodies on the mast cells or basophils. In the second phase, further exposure to allergens induces the degranulation of mast cells or basophils, leading to the secretion of inflammatory mediators, cytokines, chemokines, and eicosanoids [7]. This process causes several systemic or localized effects, such as vasodilation, smooth muscle contraction, mucous production, and tissue eosinophilic infiltration [8]. In vivo tests include the skin prick test (SPT), intradermal test, and nasal or conjunctival provocation test, whereas in vitro tests involve measurement of serum total immunoglobulin E (TIgE) and serum-specific immunoglobulin E (SIgE) levels (e.g., the fluoroallergosorbent test (FAST), ImmunoCAP (Phadia AB, Uppsala, Sweden), Immulite (Siemens Healthcare Diagnostics, Tarrytown, NY, USA), and multiple allergen simultaneous test (MAST)) [9,10].

Although SPT is commonly performed in clinics, there are several limitations in terms of a patient’s comfort, and the results may be affected by skin conditions or medications [11,12,13,14]. In contrast, in vitro tests for detecting IgE in the serum are independent of the effects of medications or skin disease [15]. Furthermore, quantitative results have the advantage of reduced subjectivity or operator error [16]. Among several in vitro test methods, the multiple allergen simultaneous test (MAST) is more widely used because it involves a simpler and faster procedure. MAST-immunoblot assay is the most recently developed method, and many studies are being conducted on it targeting allergic patients [9,17,18,19]. In addition, MAST has the advantage of being able to test many allergens at once.

However, only a few studies have investigated the allergen pattern in isolated seasonal or perennial allergic conjunctivitis patients using the MAST-immunoblot assay. Therefore, the purpose of this study is to analyze the results of the MAST-immunoblot assay in patients with isolated allergic conjunctivitis. Furthermore, through subgroup analysis, we investigated the correlation between IgE detection rate and season, sex, and age.

## 2. Materials and Methods

This study was conducted with the approval of the Institutional Review Board of the Asan Medical Center and University of Ulsan College of Medicine, Seoul, Korea (2020–1858). The study adhered to the tenets of the Declaration of Helsinki and followed the Good Clinical Practice guidelines. A retrospective chart review was performed for patients who underwent a MAST-immunoblot assay and had a diagnosis of allergic conjunctivitis.

### 2.1. Patient Enrollment

Allergic conjunctivitis was diagnosed using the following criteria: (1) clinical symptoms of ocular itching with hyperemia, foreign body sensation, ocular pain, and secretion of white mucus with or without seasonal variation; and (2) specific allergic conjunctivitis signs seen on slit lamp examination, including conjunctival hyperemia, conjunctival edema, and conjunctival follicle or papillary hypertrophy [20]. Patients were excluded from analysis using the following criteria: (1) diagnosed with IgE and non-IgE mediated ocular allergy (vernal keratoconjunctivitis and atopic keratoconjunctivitis) or non-IgE mediated ocular allergy (giant papillary conjunctivitis, contact keratoconjunctivitis); (2) allergic symptoms other than those involving the eye, such as rhinitis, dermatitis, and asthma; and (3) comorbid conditions that could affect serum IgE levels, such as infectious diseases caused by *Mycoplasma pneumoniae*, *Campylobacter jejuni*, Candida, helminth, and human immunodeficiency virus; neoplastic diseases; pregnancy; or postpartum conditions.

### 2.2. MAST-Immunoblot Assay

**Figure 1:**
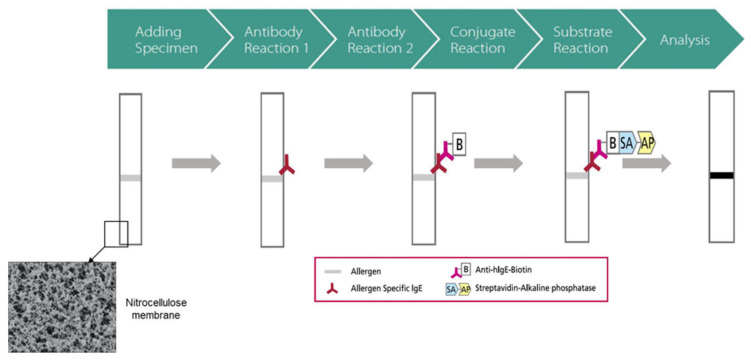
Schematic explanation of the measurement principle of the multiple allergen simultaneous test-immunoblot assay (AdvanSure AlloStation Smart II).

All patients underwent the MAST-immunoblot assay using the AdvanSure AlloStation Smart II (AS: LG Life Science, Seoul, Korea). Testing was performed according to the manufacturer’s recommendations. Briefly, 50 µL of each patient’s serum was pipetted into a reaction chamber containing the allergens on a nitrocellulose membrane and incubated at room temperature for 45 min. After washing, mouse anti-human IgE antibody coupled with biotin was added and incubated at room temperature for 30 min. Anti-human IgE antibody coupled with biotin acted as a secondary antibody, catalyzing the transformation of a specific substrate with a distinguishable property. After washing to remove the unbound antibodies, 250 mL of streptavidin conjugated to alkaline phosphatase was added and incubated at room temperature for 20 min. Non-bound conjugate was removed by washing. After adding the color-formation solution and incubating at room temperature for 20 min, test strips were completely dried. The result was converted into IU/mL through the formula for each antigen built into the program, which was measured according to the color intensity of the band (Figure 1).

MAST-immunoblot assay results were obtained for TIgE and SIgE for 57 allergens. TIgE was classified into positive and negative, using 100 IU/mL as a cut-off. If the TIgE concentration was more than 100 IU/mL, patients were included in the TIgE+ group. In this study, allergens with a SIgE concentration of 0.7 IU/mL or above were considered positive.

The patients were then classified based on the season when the eye symptoms were exacerbated: spring (March to May), summer (June to August), fall (September to November), and winter (December to February). The results for TIgE and SIgE positivity were classified into four groups according to the MAST-immunoblot assay results: Group 1 (TIgE+, SIgE+), Group 2 (TIgE−, SIgE+), Group 3 (TIgE+, SIgE−), and Group 4 (TIgE−, SIgE−). In addition, differences in the positivity of TIgE and SIgE between sex and age groups were also analyzed. Regarding age groups, patients were divided into young and old groups (≤35 years and ≥35 years). The rationale for the baseline of 35 years is based on a previous study in which serum IgE levels peaked in the late 20s (28–30 years old) and then showed declining patterns from the late 30s (36–40 years old) [21].

### 2.3. Statistical Analysis

All statistical analyses were performed using SPSS statistical software (version 25.0, IBM Corp., Armonk, NY, USA) and statistical significance was defined as *p* < 0.05. The comparison of subgroup variables classified by season when the eye symptoms were exacerbated and by IgE positivity was performed using the non-parametric Kruskal-Wallis test, Pearson’s chi-square test, and Fisher’s exact test. Comparison by sex and age groups was performed with the non-parametric Mann–Whitney U test and Pearson’s chi-square test.

## 3. Results

### 3.1. Demographic Data

A total of 120 patients (32 men, 88 women) with an average age of 35.7 ± 17.5 years (range, 4–78 years) were included in this study. When divided by season of exacerbating symptoms, 29, 49, 32, and 10 patients were included in the spring, summer, fall, and winter groups, respectively.

### 3.2. MAST-Immunoblot Assay (Overall)

The allergens that were most frequently detected through MAST in the total study population were *Dermatophagoides farinae* (36.7%), *Dermatophagoides pteronyssinus* (34.2%), *Tyrophagus putrescentiae* (17.5%), *Alternaria* (15.8%), and house dust (14.2%) (Table 1). House dust mites, such as *D. farinae* and *D. pteronyssinus* showed the highest detection rates regardless of the season; however, other allergens showed differences according to the season (spring: house dust, *Acarus siro*, and rye pollen; summer: *Alternaria*, *T. putrescentiae*, and house dust; fall: Mugwort, *T. putrescentiae*, ragweed, oxeye daisy, Japanese hop, and dandelion; and winter: *Alternaria*, house dust, cat, oxeye daisy, and Cladosporium (Table 1)).

### 3.3. Subgroup Analysis of MAST-Immunoblot Assay

**Figure 2:**
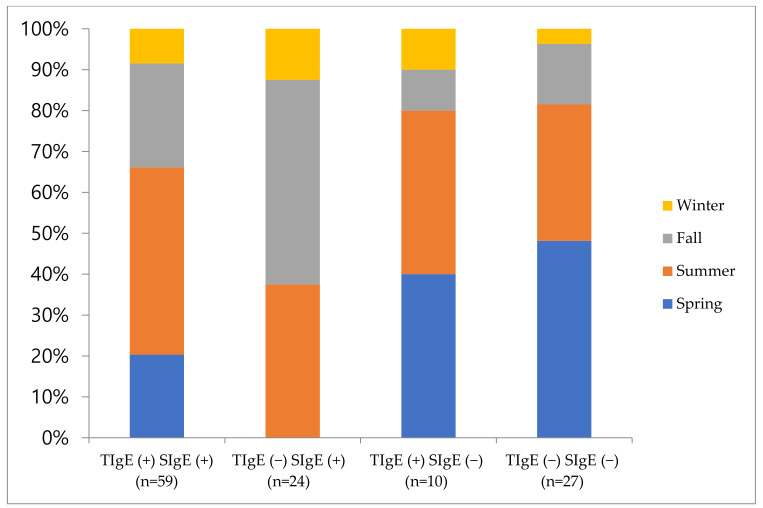
Seasonal distribution of each subgroup according to the detection of serum total immunoglobulin E and serum specific immunoglobulin E. TIgE, serum total immunoglobulin E; SigE, serum specific immunoglobulin E.

Table 2 shows the comparison results between subgroups according to the detection of TIgE and SIgE. Sixty-nine patients (57.5%) and 83 patients (69.2%) were positive for TIgE and SIgE, respectively, and 59 patients (49.2%) were positive for both TIgE and SIgE. Ninety-three patients (77.5%) showed either TIgE or SIgE positivity, and neither was detected in 27 patients (22.5%). There was a significant difference between subgroups with regard to the sex ratio (*p* = 0.002), but not age (*p* = 0.154) (Table 2). There was a significant difference in the sex ratio between Groups 1 and 4 (*p* = 0.001), and between Groups 3 and 4 (*p* = 0.030). Although there was no significant difference regarding age, Group 1 was younger than the rest of the groups. Figure 2 shows the results for seasonal distribution of allergen sensitizations.

### 3.4. Effect of Sex and Age on MAST-Immunoblot Assay

Positive detection rates for TIgE and SIgE were higher in men than in women (TIgE: 84.4% for men vs. 47.7% for women, and SIgE: 81.2% for men vs. 64.8% for women); however, only the differences in TIgE were statistically significant (*p* < 0.001) (Table 3). Although there was no statistically significant difference in SIgE, men had a higher detection rate (*p* = 0.084). In addition, 96.9% of men were positive for either TIgE or SIgE, showing a significant difference compared to women (70.5%) (*p* = 0.001) (Table 3). According to the subgroup analysis with regard to age, positive detection rates for TIgE and SIgE were higher in the young group than in the old group (TIgE: 64.3% for young group vs. 48.0% for old group and SIgE: 77.1% for young group vs. 58.0% for old group), but only the differences in SIgE were statistically significant (*p* = 0.025) (Table 3). Although there was no statistical significance in TIgE, the young group had a high detection rate (*p* = 0.075) (Table 3).

## 4. Discussion

Identification of the sensitizing allergen is important in diagnosis, avoidance, and allergy treatment, especially before initiating specific immunotherapy [22]. The effect of immunotherapy on relieving ocular symptoms has been shown in previous studies [23,24]. In particular, even several years after the completion of immunotherapy, its efficacy for ocular symptoms remains [25]. The widely used method for detecting the sensitizing allergen is the SPT, which has both high sensitivity and good reproducibility. However, individuals with a high risk for anaphylaxis are contraindicated to undergo SPT. Medications may affect the results of SPT when taken concurrently, including antihistamines, tricyclic antidepressants, omalizumab, and topical steroids in the test area [11,12,13].

Various test methods have been developed to overcome these limitations, among which, the detection of serum IgE, which acts as a key mediator in the occurrence of allergy, is the mainstream [26]. The radioallergosorbent test (RAST), FAST, and MAST have been developed for detecting IgE in serum. MAST is mainly used because of its advantages such as being free from isotope, faster test time, low cost, and ability to simultaneously test for multiple allergens. Recently, the MAST-immunoblot assay was developed; which reduces the testing time from 48 h to less than 3 h and involves a simplified testing procedure compared to the previous MAST-chemiluminescence assay [17]. Moreover, it requires a lesser amount of blood sample. The MAST-immunoblot assay has shown similar or better allergen detection rates compared to SPT or other in vitro IgE methods [18].

In this study, house dust mites, such as *D. farinae* (36.7%) and *D. pteronyssinus* (34.2%), were the most detected allergens. This is consistent with the results of a large-scale study of Korean patients with allergic disease [18,27]. In addition, house dust mite showed the highest detection rates regardless of the season, which is in line with the fact that the most common cause of perennial allergic conjunctivitis is house dust mite [28]. There was a seasonal variation when the detection rate is was analyzed by season, indicating that seasonal exposure to different allergens occurred, leading to the development of seasonal allergic conjunctivitis or an exacerbation of perennial allergic conjunctivitis. In spring, the positive detection rate of *A. siro* (13.8%) was higher than that in other seasons, possibly due to the peak of the *A. siro* population in spring (especially April) [29]. In the life cycle of *T. putrescentiae*, a high temperature and humid environment are essential; therefore, the detection rate is expected to be high in summer and autumn (22.4% and 21.9%, respectively, in our study). The high detection rate of *Alternaria* (24.5%) in summer can be explained by similar reasons. Mugwort (31.3%), ragweed (15.6%), Japanese hop (15.6%), and dandelion (15.6%) had a higher positivity rate in fall than in other seasons, possibly due to the abundance of pollen in the air during fall [30]. In winter, the positivity for house dust and cat allergen was high, which can be explained by the relatively lower ventilation owing to changes in climate. However, the high detection rate of *Alternaria* and *Cladosporium* was not in line with their life cycles.

The percentage of individuals who were positive for TIgE or SIgE for at least one allergen was 57.5% and 69.2%, respectively. This result is similar to the results of previous studies using the MAST-immunoblot assay, but lower than the results of SPT [31,32]. In vitro methods measure circulating IgE in serum; however, in practice, most IgE is attached to the mast cells of the tissue; therefore, the IgE level can be underestimated by in vitro methods (lower sensitivity, false negativity) [5,33]. Conversely, in in vivo methods, the positive detection rate increases due to non-IgE-mediated triggers or alternative mechanisms (lower specificity, false positivity) [5,34]. There were 8.3% (*n* = 10) of patients with increased TIgE levels, although no SIgE was detected. Some interpret this as involvement of other factors that increase the serum IgE levels except allergy, such as parasitic infection, neoplastic diseases, and immune deficiencies [35]. However, we excluded patients with these diseases from the study, and regardless of sensitization to SIgE, an increase in TIgE concentration was found to be associated with the onset and worsening of allergy symptoms [36]. Both TIgE and SIgE were negative in 22.5% of patients. It is unclear how many of these patients may have had local allergic conjunctivitis which cannot be detected through SIgE. In addition, considering that 0.7 IU/mL was used as a cut-off for positive detection of SIgE, different results may be obtained if the cut-off value is changed to 0.35 IU/mL. Moreover, underestimated IgE levels in in vitro tests may also result in differences in the test results [5,33]. When seasonal distribution between subgroups was analyzed, the proportions of positive cases in each season were different. In this study, there was a difference in the TIgE or SIgE positivity according to sex. However, it was significant only for TIgE. In a prior study, the National Health and Nutrition Examination Survey 2005–2006 of the US population, men were more likely to exhibit positive SIgE tests and elevated levels of SIgE than women [37]. Some studies have suggested that differences in the production of IgE may be associated with differences in genetic, hormonal, and environmental factors involved in IgE control [38,39]. Although no statistical significance was observed in this study, it was found that men had a tendency of showing a higher SIgE positivity rate than women. Therefore, this needs to be confirmed in a larger study.

With regard to the positivity rate of TIgE or SIgE, there was a difference according to age. The SIgE positivity rate was significantly higher in the young group than in the old group; however, TIgE did not show any significant difference. Several studies have shown that serum IgE level is higher in younger than in older individuals [37,40,41]. The decline in the prevalence of IgE sensitization in older individuals may be attributed to gradual deterioration of the immune system, called “immunosenescence” [42,43]. The secretion of IgE depends on the interaction between B and T lymphocytes and is reduced by the naturally occurring involution of the thymus, which plays a key role in the development of T lymphocytes [44]. In an animal study, the transplantation of thymocytes in young mice did not cause a change in IgE secretion, whereas in aged mice, IgE secretion was triggered to a level similar to that in young mice [45]. A large-scale study reported that SIgE levels decreased with aging and that TIgE levels were maintained despite aging. Furthermore, TIgE levels gradually increased after 60 year-old and peaked in the oldest subgroup of 85 years or older, which is attributed to the impaired regulatory function with aging [21].

This study has several limitations. First, the number of patients included in the study was relatively small; however, it was challenging to identify patients with isolated allergic conjunctivitis who did not have associated allergic rhinitis, asthma, or dermatitis. Second, other factors known to influence the IgE level (socioeconomic state, education level, and residual area) were not analyzed. Third, because the study was performed in a single region, the results may not reflect local differences. Finally, since SPT was not concurrently performed in this study, the correlation with allergens detected in the MAST-immunoblot assay could not be analyzed.

## 5. Conclusions

When detecting the sensitizing allergen in patients with isolated allergic conjunctivitis using the MAST-immunoblot assay, allergen-triggered sensitization differed according to the season when the eye symptoms were exacerbated. Furthermore, IgE detection differed according to sex and age. This is the first study to perform the MAST-immunoblot assay by selecting only patients with no other allergic symptoms except ocular symptoms, while targeting only IgE-mediated allergic conjunctivitis. Since the results differed according to sex and age, the MAST results should be interpreted carefully in patients with isolated allergic conjunctivitis. We expect that our study results can be used by clinicians when predicting the pattern of allergens according to seasons and interpreting test results according to age and sex.

## Figures and Tables

**Table 1:** Positive rate of each allergen-specific immunoglobulin E (IgE) detected by multiple allergen simultaneous test-immunoblot assay subdivided by seasons.

Allergen	Total (100%, *n* = 120)	Spring (24.2%, *n* = 29)	Summer (40.8%, *n* = 49)	Fall (26.7%, *n* = 32)	Winter (8.3%, *n* = 10)
TIgE	57.5	51.7	63.3	50.0	60.0
*D. farinae*	36.7	27.6	46.9	31.3	30.0
*D. pteronyssinus*	34.2	17.2	44.9	34.4	30.0
*Tyrophagus putr.*	17.5	6.9	22.4	21.9	10.0
*Alternaria*	15.8	3.4	24.5	12.5	20.0
House dust	14.2	17.2	16.3	6.3	20.0
Mugwort	11.7	3.4	6.1	31.3	0.0
Cat	10.8	3.4	14.3	9.4	20.0
Ragweed	9.2	3.4	8.2	15.6	10.0
Oxeye-daisy	8.3	0.0	6.1	15.6	20.0
Japanese hop	8.3	3.4	6.1	15.6	10.0
Birch-Alder mix	8.3	6.9	6.1	12.5	10.0
Rye pollen	7.5	10.3	4.1	9.4	10.0
Dandelion	6.7	0.0	4.1	15.6	10.0
*Acarus siro*	5.8	13.8	4.1	0.0	10.0
Milk	5.0	6.9	6.1	3.1	0.0
Cedar, Japan	5.0	3.4	8.2	3.1	0.0
*Cladosporium*	5.0	0.0	4.1	6.3	20.0
Russian Thistle	4.2	0.0	6.1	6.3	0.0
Egg white	4.2	0.0	2.0	9.4	10.0
Cockroach	3.3	0.0	6.1	3.1	0.0
Peach	3.3	3.4	4.1	0.0	10.0
Mackerel	3.3	0.0	2.0	9.4	0.0
Sycamore mix	3.3	0.0	4.1	3.1	10.0
Pigweed	3.3	0.0	6.1	3.1	0.0
Bermuda grass	2.5	0.0	2.0	3.1	10.0
Sweet vernal grass	2.5	3.4	2.0	0.0	10.0
Dog	2.5	6.9	2.0	0.0	0.0
Orchard grass	2.5	3.4	4.1	3.1	0.0
Hazelnut	2.5	3.4	0.0	3.1	10.0
Acacia	2.5	0.0	2.0	3.1	10.0
Sallow willow	2.5	3.4	2.0	0.0	10.0
CCD mix	2.5	3.4	2.0	3.1	0.0
Reed	2.5	3.4	2.0	0.0	10.0
Timothy grass	2.5	3.4	4.1	3.1	0.0
Oak, White	2.5	6.9	0.0	0.0	10.0
Crab	1.7	0.0	2.0	3.1	0.0
Honey bee	1.7	0.0	2.0	0.0	10.0
Potato	1.7	0.0	2.0	0.0	10.0
Soybean	0.8	0.0	2.0	0.0	0.0
Pine	0.8	0.0	0.0	0.0	10.0
Poplar mix	0.8	0.0	0.0	0.0	10.0
Pork	0.8	3.4	0.0	0.0	0.0
*Aspergillus*	0.8	0.0	0.0	3.1	0.0
Cucumber	0.8	0.0	2.0	0.0	0.0
Wheat flour	0.8	0.0	2.0	0.0	0.0
Penicillium	0.8	0.0	2.0	0.0	0.0
Latex	0.8	0.0	2.0	0.0	0.0
Cacao	0.8	0.0	2.0	0.0	0.0
Pupa	0.8	0.0	0.0	3.1	0.0
Mango	0.8	0.0	2.0	0.0	0.0
Banana	0.0	0.0	0.0	0.0	0.0
Raw chestnut	0.0	0.0	0.0	0.0	0.0
Buckwheat meal	0.0	0.0	0.0	0.0	0.0
*Candida albicans*	0.0	0.0	0.0	0.0	0.0
Guinea pig	0.0	0.0	0.0	0.0	0.0
Peanut	0.0	0.0	0.0	0.0	0.0
Sesame	0.0	0.0	0.0	0.0	0.0

IgE, immunoglobulin E; TIgE, serum total immunoglobulin E; *D. farinae*, *Dermatophagoides farinae*; *D. pteronyssinus*, *Dermatophagoides pteronyssinus*; *Tyrophagus putr.*, *Tyrophagus putrescentiae*; CCD, Cross-reactive carbohydrate determinant.

**Table 2:** Comparison of the test results between subgroups according to the detection of TIgE and SIgE.

	Number of Patients (%)	Age (Years)	M:F
Group 1; TIgE (+) SIgE (+)	59 (49.2)	33.6 ± 18.2	22:37
Group 2; TIgE (−) SIgE (+)	24 (20.0)	34.2 ± 16.3	4:20
Group 3; TIgE (+) SIgE (−)	10 (8.3)	42.1 ± 16.0	5:5
Group 4; TIgE (−) SIgE (−)	27 (22.5)	39.1 ± 17.2	1:26
*p* value		0.154	0.002

MAST, multiple allergen simultaneous test; TigE, serum total immunoglobulin E; SigE, serum specific immunoglobulin E.

**Table 3:** Comparison of the test results between sex and age groups.

Sex (Male vs. Female)	Total (*n* = 120)	Male (*n* = 32)	Female (*n* = 88)	*p* value
Age	35.7 ± 17.5	33.3 ± 16.9	36.55 ± 17.7	0.345
TIgE(+):TIge(−)	69:51 (57.5%:42.5%)	27:5 (84.4%:15.6%)	42:46 (47.7%:52.3%)	<0.001
SIgE(+):SIge(−)	83:37 (69.2%:30.8%)	26:6 (81.2%:18.8%)	57:31 (64.8%:35.2%)	0.084
AIgE(+):AIge(−)	93:27 (77.5%:22.5%)	31:1 (96.9%:3.1%)	62:26 (70.5%:29.5%)	0.001
**Age (young vs. old)**	**Total (*n* = 120)**	**Young group** **(*n* = 70)**	**Old group (*n* = 50)**	***p* value**
Sex (male:female)	32:88	20:50	12:38	0.577
TIgE(+): TIge(−)	69:51 (57.5%:42.5%)	45:25 (64.3%:35.7%)	24:26 (48.0%:52.0%)	0.075
SIgE(+): SIge(−)	83:37 (69.2%:30.8%)	54:16 (77.1%:22.9%)	29:21 (58.0%:42.0%)	0.025
AIgE(+):AIge(−)	93:27 (77.5%:22.5%)	58:12 (82.9%:17.1%)	35:15 (70.0%:30.0%)	0.096

TIgE, serum total immunoglobulin E; SIgE, serum specific immunoglobulin E; AIgE(+), positive for at least one of TIgE and SIgE; AIgE(−), negative for both TIgE and SIgE.

## Data Availability

Data are available upon request from the authors.
